# Functional, rheological, and microstructural properties of hydrothermal puffed and raw amaranth flour suspensions

**DOI:** 10.1002/fsn3.2970

**Published:** 2022-07-19

**Authors:** Tanja Lux (neé Bantleon), Martha Kardell, Frederike Reimold, Adam Erdoes, Eckhard Floeter

**Affiliations:** ^1^ Department of Food Processing Technology Technische Universität Berlin, Institute for Food Technology and Food Chemistry Berlin Germany; ^2^ Institute for Agricultural and Urban Ecological Projects (IASP) Affiliated to Humboldt Universität Berlin Berlin Germany; ^3^ University of Applied Sciences Bremerhaven, Food Technology of Animal Products Bremerhaven Germany

**Keywords:** color change, pasting properties, pseudocereal suspension, puffed amaranth, water absorption

## Abstract

The pseudocereal amaranth is commonly used in food as whole puffed grain. To improve the utilization of amaranth, hydrothermally treated suspensions of puffed and raw *Amaranthus caudatus* flour and their blends were investigated in this study. The suspensions were hydrothermally treated at 20, 50, and 80°C for 1, 5, and 24 h. The blends were treated at 80°C for 1 h. The effect of hydrothermal treatments of the suspensions on their morphological (color, SEM), water‐binding, and rheological‐functional properties was studied. The puffed amaranth suspensions exhibited cold swelling properties by rapid viscosity increase and significant water absorption properties. It was found that hydrothermal treatment at 80°C for 1 h significantly increased water absorption and viscosity in puffed and raw flour suspensions. However, the puffed suspensions showed significantly higher values in water binding and viscosity. Suspensions of raw amaranth flour showed increasing color differences with increasing temperature. Blends of raw and puffed amaranth flour resulted in a decreasing color change with increasing puffed flour content. Water absorption of the samples increased with an increasing puffed flour content. Raw amaranth flour and the 50/50 (puffed/raw) blend had the lowest, 10/90 and 20/80 (puffed/raw), and showed similar viscosity profiles to suspensions of pure puffed flour.

## Graphical abstract

To improve the utilization of amaranth, hydrothermally treated suspensions of puffed (PAF) and raw (AF) *Amaranthus caudatus* flour and their blends were investigated. The puffed amaranth suspensions showed cold swelling properties through rapid viscosity increase without temperature influence. In the blends of PAF and AF, PAF had a stronger effect on viscosity. A minor addition of PAF significantly increased the viscosity of AF (>68 °C). Increasing the amount of PAF in the blends resulted in an increase in water absorption.

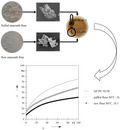

## 1 INTRODUCTION

The production of gluten‐free food is still a challenge for the food industry and science. The ingredients and functional properties of gluten‐free cereals such as rice or corn often do not meet nutritional requirements. Amaranth is a pseudocereal with high nutritional properties suitable for gluten‐free dietary products (Martinez‐Villaluenga et al., 2020). However, its potential is currently not fully exploited, for example, amaranth is not yet as widespread or known as quinoa. When amaranth is used, it is often in its puffed form, as this results in sensory changes that have a positive effect on the pseudocereals (Schmidt et al., 2021). For centuries, puffing on a hot plate has been one of the oldest and most traditional preparations for amaranth grains. This is a simple, inexpensive, and fast process that requires temperatures above 190°C for 15–30 s (Singh & Sood, 2021). There are some foods that contain amaranth, but mainly in puffed form. For example, in India or Mexico, blends of puffed amaranth seeds and molasses are a traditional confection (Konishi et al., 2004), or the puffed seeds are used in breakfast cereals and ready‐to‐eat products (Paucar‐Menacho et al., 2018). Puffing with dry heat evaporates the water contained in the starch matrix of amaranth seeds. The starch inside the amaranth seed initially boils and eventually expands due to the steam generated. The steam, which is concentrated in the pores of the starch grains, increases the temperature and pressure. At this stage, the amaranth starch granules begin to swell, breaking the pericarp and causing the starch granules to expand. The endosperm of the amaranth seed turns into a solidified, spongy matrix formed by the spontaneous evaporation of water (Paucar‐Menacho et al., 2018). During puffing, the starch is partially gelatinized. The perisperm gives rise to a spongy matrix with 4–8‐fold increase in volume. The brownish seed coat adheres loosely to the whitish perisperm, while the embryo remains intact (Schoenlechner, 2017; Singh & Sood, 2021).

To date, researchers have focused on the development of different puffing methods (Castro‐Giráldez et al., 2012; Konishi et al., 2004; Lara & Ruales, 2002) and the associated changes in the chemical and nutritional composition of puffed amaranth (Amare et al., 2015; Burgos et al., 2018; Burgos & Armada, 2015, 2021). There are a few known research studies on hydrated raw and puffed amaranth and its applications. A study on this topic by González et al. (2002) investigated, among others, the effects of hydrated, puffed, dehulled and degerminated amaranth flours for use in precooked flour. This study shows that puffed amaranth flours (PAF) can be used in various food preparations due to its instant swelling properties. Puffing makes amaranth flour and starch soluble in cold water, which in turn increases the viscosity and water absorption of suspensions prepared from this material (González et al., 2002). In addition, puffing produces a distinct nutty and toasty flavor. All of this suggests that PAF suspensions are suitable for use in many food products such as pasta (Islas‐Rubio et al., 2014) or as an admixture in bread (Martinez‐Villaluenga et al., 2020).

In this context, the aim of this study was to investigate suspensions of raw amaranth flour (AF) and PAF with regard to the influence of time and temperature on this process. The temperature of the suspensions was chosen above the gelatinization temperature of the raw amaranth grain Amaranthus caudatus, which ranges from 62°C to 68°C (Gamel et al., 2005; Srichuwong et al., 2017), depending on the available water. In addition, different blends of amaranth flour and PAF were studied. Scanning electron microscopy (SEM), water absorption, color changes, and shear stress profiles were used to study the suspensions. The data obtained in this study can be used in the bakery and pasta industries to develop new product flavors and gluten‐free products.

## 2 MATERIALS AND METHODS

### 2.1 Raw material and sample preparation

Mature grains of *Amaranthus caudatus* Oscar blanco harvested commercially in Peru, Cotahuasi by De Guste Group Sac. were used in this study. Moisture content of the dried grains was between 12.0% and 13.0%. Amaranth seeds were cleaned and stored in a cold (approximately 15°C), dark, and dry place for no more than half a year. In this study, PAF and AF, as well as blends thereof, were used.

Puffing was conducted on a hot plate at atmospheric pressure. For this purpose, approximately 100 g of the amaranth grains were placed in a 200°C heated pan and stirred. After 10–15 s, most of the grains puffed, turned white and were removed from the pan. Using a sieve with mesh size of 1.0 mm, burnt, and not puffed seeds were excluded.

Amaranth flour and PAF were milled at the finest grinding level with a grain mill attachment for a stand mixer (KitchenAid Europe). AF and PAF were stored in polyethylene bags at −18°C until use. Blends were prepared with decreasing PAF from 100% to 0% of the total flour on the day of analysis.

To show the particle size distribution of the AF and PAF, a sieve analysis was performed with vibratory sieve shaker AS 200 Basic (Retsch GmbH). PAF and AF samples were applied to a sieve stack that complied with ASTM Standard E11:87 mesh size of 1000 μm (No. 18), 500 μm (No. 35), 250 μm (No. 60) and 180 μm (No. 80) as well as a sieve bottom for particles <180 μm. 200 ± 0.1 g of PAF and AF were sieved for 5 min by sonic pulsing of 3000 min^−1^ (60 Hz). The fractions on each sieve were calculated as percentage of dry basis. Three different grinding batches were each investigated (*n* = 3).

### 2.2 Hydrothermal treatment of samples

The hydrothermal treatment of the amaranth samples was carried out in covered glass beakers at a constant temperature shaking water bath Precision SWB 25 (Thermo Fisher Scientific Inc.), controlled within ±0.5°C, and were shaken at 30 rpm to achieve sufficient moisturizing. For each assay, 10 ± 0.05 g of PAF, AF or blends thereof were placed in 100 g deionized water (1:10, w/v). Hydrothermal temperatures include treatments at room temperature (20°C), temperatures below (50°C), and above the gelatinization temperature (80°C) of amaranth starch. Hydrothermal treatment temperatures were conducted for 1, 5, and 24 h for PAF and AF, and for blends at 80°C for 1 h. After completion of each hydrothermal process, the samples were cooled and analyzed at 20°C. The temperatures and time intervals studied were taken from a previously conducted study (Lux et al., 2021). All hydrothermal suspensions were run three times (*n* = 3) and analyzed at least twice.

### 2.3 Scanning electron microscopy (SEM)

The SEM (Hitachi S 2700, Hiatchi) located at Technische Universität, Berlin was used to study the changes in microstructure within PAF and AF during hydrothermal processes (1, 5, 24 h for 20, 50, 80°C). For preparation, soaked amaranth samples were air dried, placed on aluminum sample plates and coated with gold. An acceleration potential of 20 kV and a vacuum of 10^−4^ mbar was used for making microscopic images. PAF and AF samples were investigated raw and hydrated at 50 and 80°C for 1 h, respectively.

### 2.4 Water absorption index (WAI)

Water absorption index of the suspensions was determined according to Schwenke et al. (1981) with minor modifications: 1.5 ± 0.01 g of the dry materials (PAF, AF, blends) were added in 50 ml Eppendorf tubes and hydrated with 15 g deionized water. The suspensions were mixed by vertexing for 20 sec to ensure complete moisturizing. Afterwards, the samples were hydrothermally treated at 20, 50, and 80°C for 1, 5, and 24 h. The hydrated PAF and AF suspensions were centrifuged at 14,000 ×*g* for 10 min, at 20°C (Heraeus Multifuge 3SR+, Thermo Fisher). The supernatant was discarded and weighted. The WAI is calculated from g_water_/g_sample_. All hydrothermal suspensions were conducted three times (*n* = 3) and analyzed at least twice.

### 2.5 Color determination

Color values of the hydrothermal treated PAF and AF suspensions and blended suspensions were determined using a Chroma‐Meter CR 200 (Konica Minolta Sensing Europe B.V.). CIELAB parameters (L*a*b*) were measured with D65 illumination conditions. Before color measurement, a white standard plate was used for calibration. For each measurement approximately 20 ml of the samples was placed in a flat‐bottomed transparent cylinder with a diameter of 20 mm. The measurement was performed *n* = 5 for each sample.

From measured data, the color difference (∆E, Equation [1]) (Tayefe et al., 2020) of untreated and prepared samples and yellowing index (YI) were calculated using the following Equation (2) (Rufián‐Henares et al., 2006):

(1) $$∆E=\sqrt{\left({\left(∆L^{*}\right)}^{2}+{\left(∆a^{*}\right)}^{2}+{\left(∆b^{*}\right)}^{2}\right)}$$

(2) $$\mathrm{YI}=142.86\;b^{*}/L^{*}$$

L*‐value for brightness,

a*‐value for red/green opponents,

b*‐value for blue/yellow opponents.

### 2.6 Rheological measurements

A controlled shear rate test was carried out to investigate the viscosity changes of the thermally treated samples. A Physica MCR 302 rheometer (Anton Paar GmbH) with CC 27/P1 concentrical cylinder measuring unit (DIN standard) was used. The shear rate was increased from 0.1 to 100 s^−1^ continuously in 3.3 min at 20°C. All samples were measured in triplicates.

To characterize the rheological behavior of the thermally treated samples, the flow curves were described with the Herschel‐Bulkley mathematical model function. This model describes the data of pseudoplastic (*n* < 1), dilatant (*n* > 1), or Newtonian fluids (*n* = 1) considering a possible yield stress. The regression model is described with the following formula (3) (Rao, 2007):

(3) $$\text{Herschel}-\text{Bulkley}:\tau =\tau_{0}+k*{\dot{\gamma }}^{n}$$

*τ*: shear stress (Pa).

*τ*_0_: yield stress (Pa).

*k*: consistency coefficient (Pa s^*n*^).

$\dot{\gamma }:$shear rate (s^−1^).

*n*: flow index (dimensionless).

### 2.7 Statistical analysis

The experimental design was a completely randomized 2^3^ factorial design with at least three replications. The statistical analysis was carried out with SigmaPlot (Sigmaplot 11.0 for windows, Systat Software Inc.). Significant differences between the samples were calculated using the analysis of variance (ANOVA). Mean values were compared by Tukey's test with *p* < .05. Results are shown with the mean value including the standard deviation of mean. Rheological data were analyzed using the Rheoplus software (Anton Paar GmbH). Each measurement was conducted at least in duplicate.

## 3 RESULTS AND DISCUSSION

### 3.1 Sieve analysis and SEM of PAF and AF

**FIGURE 1:**
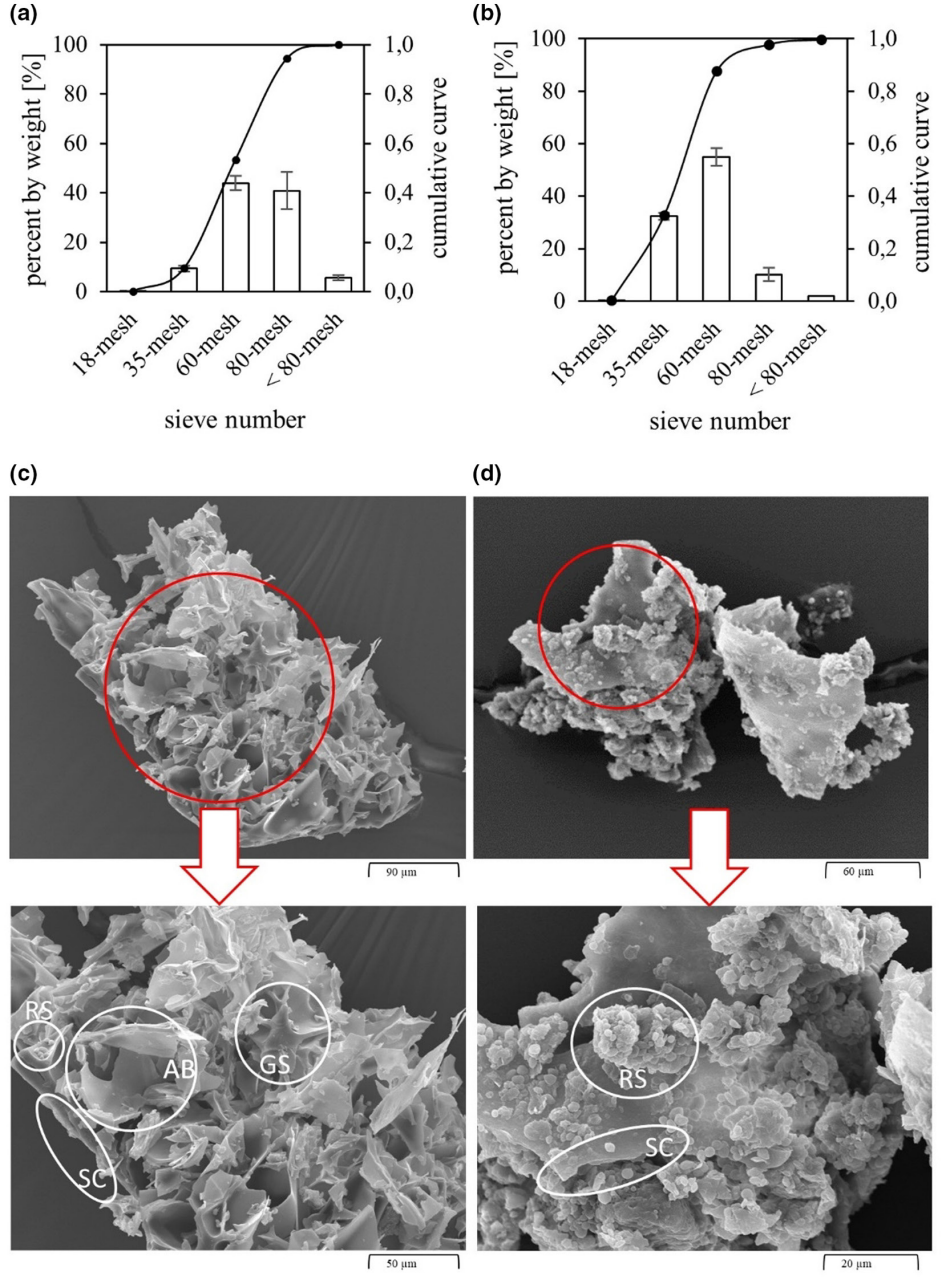
Particle size distribution of (a) puffed amaranth flour (PAF), (b) raw amaranth flour (AF), and (c) Scanning Electron Micrographs (SEM) of (c) PAF and (d) AF. *AB*, air bubble of puffed amaranth; *GS*, gelatinized starch; *RS*, raw starch granules; *SC*, seed coat. Values are given as mean ± SD. *n* = 6

The particle size distribution is an important quality parameter for flours that can influence, for example, the flow behavior of suspensions. The quantity distribution of the sieve analysis of PAF showed the largest proportion between mesh sizes 60, corresponding to a particle size >250 μm (43.9%) and 80, corresponding to a particle size >180 μm (40.9%) (Figure 1a). The particle size distribution of sieved AF is coarser, as evidenced by the higher proportion of 60‐mesh (55.0%) and 35‐mesh (32.4%). This corresponds to a main particle size of >250 μm and >500 μm of the amaranth flour, respectively. The study of Alonso‐Miravalles et al. (2020) reported that the flours of the pseudocereals amaranth and quinoa had irregular particles. Further, they reported clusters of starch granules. In the present study, such irregular structures and starch granules clusters were also observed in AF (Figure 1d). Comparison of the two milled materials shows that PAF was milled finer and has a more homogenous particle size, shown by the narrower particle size distribution. This is due to the differences in appearance of the two raw materials, which can be seen in the SEM in Figure 1c,d. As expected, puffing changes the structure of the amaranth grains and thus also of the flour. The particles of puffed amaranth flour exhibit both raw starch grains (RS) and gelatinized starch (GS). In addition, the air bubbles burst due to milling and the seed coat can be seen. The raw PAF sample without hydrothermal treatment has a spongy structure resulting from the puffing process (Figure 1c). Due to the spongy structure and trapped air, there is little resistance to milling, resulting in smaller particle sizes (Hidalgo et al., 2016). By contrast, raw amaranth grains have a compact structure that offers more resistance during milling, resulting in larger particles. Raw amaranth flour exhibited larger seed coat fragments (SC) with single (diameter: 0.5–2.0 μm) or agglomerated RS (Figure 1d). This is consistent with the findings from Burgos and Armada (2021), who described similar observations with different cereal grains before and after puffing. As Burgos and colleagues pointed out, the starch granules completely changed their shape due to puffing. The original structure of the starch can no longer be seen, indicating that this component has been altered by heat exposure. They explain this by a dextrinization of the starch resulting from puffing. This is due to the high temperature, low humidity, and high pressure. The particle size and distribution of the flour is an important factor on which properties such as WAI and rheological behavior depend and affects hydration properties (Islas‐Rubio et al., 2014). A narrow distribution of particle sizes is desirable for homogenous hydration (Barbosa‐Cánovas et al., 2005). The sieve analysis showed that the particle size distribution of PAF was more homogenous than that of AF. This will further influence the properties of the PAF and AF.

### 3.2 SEM of hydrothermally treated amaranth suspensions

**FIGURE 2:**
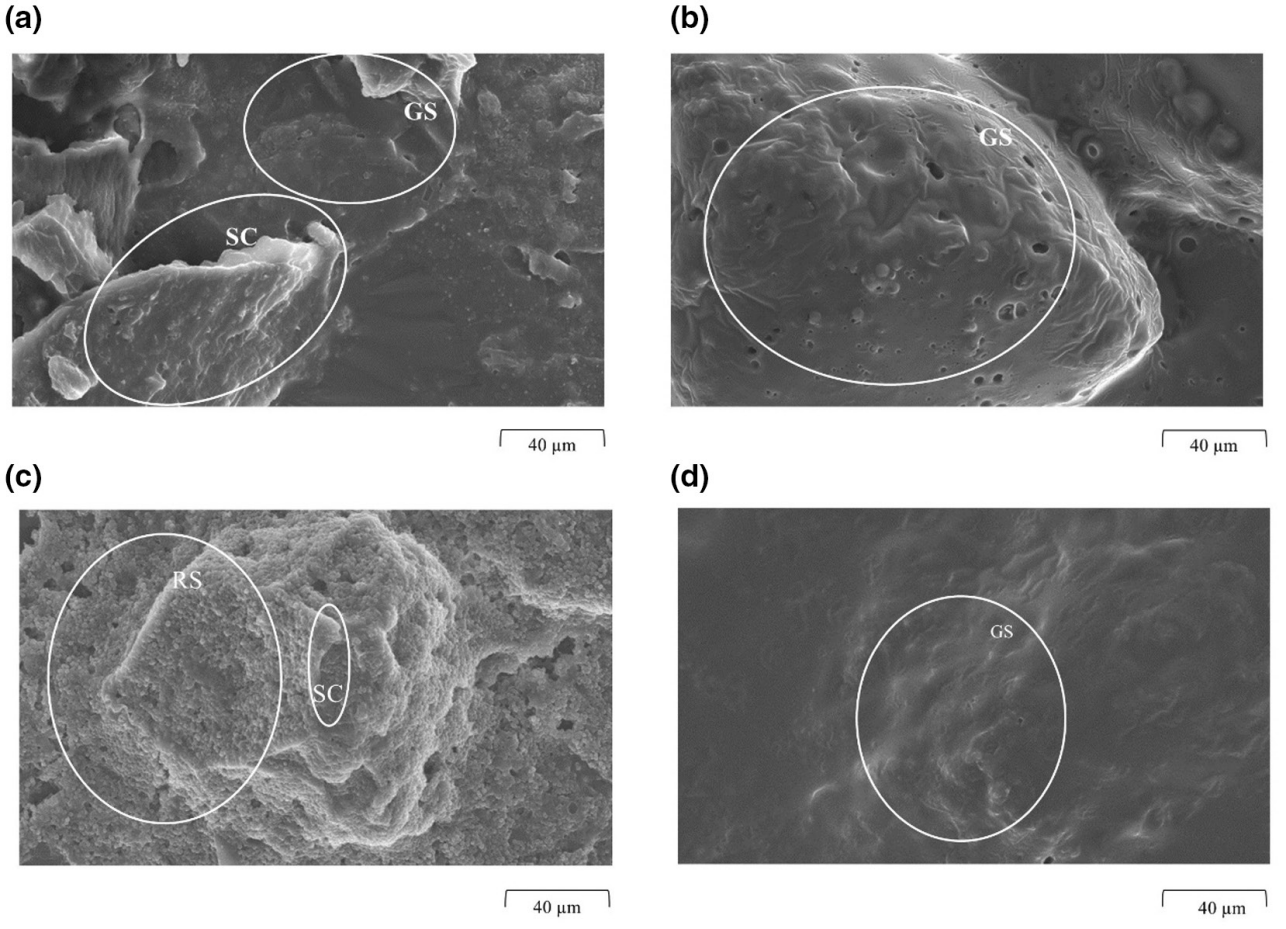
Scanning Electron Micrographs of a hydrothermally treated puffed amaranth suspension (PAF) (a) at 50°C (b) at 80°C; hydrothermally treated raw amaranth suspensions (AF) (c) at 50°C, (d) at 80°C. The swelling time was 1 h. *SC*, seed coat; *GS*, gelatinized starch; *RS*, raw starch

Scanning electron microscopy (SEM) images were used to study the morphological changes of PAF and AF during the different hydrothermal treatments. They also contribute to the explanation of further analyses. The samples were hydrated at 50°C and 80°C for 1 h. At these hydrothermal conditions, most changes occurred within the PAF and AF samples. Among other things, the treatment of the puffing changes above all the water absorption of the flour. The reorganization of the outer shell and the high porosity of the matrix are responsible for the rapid hydration of the flours. As Mariotti et al. (2006) also noted, water absorption occurs within a few seconds. For SEM of the PAF samples, at 50°C and 80°C for 1 h, individual parts of the puffed amaranth seed coat are covered with a GS (Figure 2a,b). The starch is gelatinized or damaged by puffing and makes the flour more hygroscopic. Therefore, it swells at temperatures below the gelatinization temperature of 50°C forming a homogenous suspension and starch film. This behavior was also observed in the study of González et al. (2002) and Mariotti et al. (2006), who also investigated the cold swelling properties of puffed flours. By contrast, intact starch granules were visible (RS) after AF treatment at 50°C for 1 h (Figure 2c), that is, no starch gelatinization occurred. As marked and seen in Figure 2c, these starch grains of *Amaranthus caudatus* have an average size of 0 μm to 210 μm (Singh et al., 2014). However, at 80°C and 1 h, the starch is visibly gelatinized (GS), by a homogenous starch film covering other grain components (SC) (Figure 2d). This is consistent with Bressani (1994), who showed that all starch was gelatinized when amaranth was cooked for 1 h. It is known that amaranth starch gelatinizes from a temperature of about 62°C, depending on water availability (Srichuwong et al., 2017). However, the SEM image of AF treated at 80°C for 1 h (Figure 2d), can be compared with that of PAF 50°C and 80°C, 1 h (Figure 2a,b). In all three images, it can be seen that a starch film has settled over the insoluble flour particles (e.g., embryo and seed coat particles). Since gelatinization of strach occured both at a temperature of 80°C for raw flour mixed with water and during puffing due to the influence of the high temperatures, it can be assumed that this occurred here.

### 3.3 Water absorption index of amaranth suspensions

Water absorption index indicates how much water the respective samples have absorbed after hydrothermal treatment. It can be used as a gelatinization index for raw flours and as interaction between starch and protein for precooked flours (Burgos & Armada, 2015). The WAI values are shown in Table 1. Water absorption has been drastically altered by the puffing of amaranth seeds. The outer layers of the seed are reorganized and the high porosity of the matrix accelerated its hydration (Hidalgo et al., 2016). This process was also observed in the present samples and results, and can be supported by the SEM images in Figure 1c. The interaction between time and temperature was significant for PAF WAI (*p* = .015) and for AF (*p* < .001). Comparing the WAI values of PAF and AF at the same treatment temperature, the WAI of the PAF samples was three times higher. This is consistent with the study by Hidalgo et al. (2016), who found that water absorption in puffed wheat grains had more than doubled. The strong water absorption can be explained by the structure of the puffed flours, which are shown in Figure 1c. The gelatinized starch and denaturation of proteins by puffing resulted in rapid water absorption, which is very similar to the gelatinization of starch by heat (Burgos & Armada, 2021; Gamel et al., 2005). Hidalgo et al. (2016) also found that puffed cereals absorbed most of the water within a short time. As seen from the SEM images, hydrothermal treatment of PAF at 50°C for 1 h (Figure 2a) already resulted in similar structures as treatment of AF at 80°C for 1 h (Figure 2d). A significant increase of WAI in PAF samples after 1 h was observed during all hydration temperatures (*p* < .001). This can be attributed to the swelling of the granules to many times their original size, which is due to loss of crystalline order and the absorption of water (Burgos & Armada, 2015). The highest WAI value was obtained for the PAF sample treated at 80°C for 1 h. This indicates that although a large proportion of the starch granules was gelatinized during puffing, native starch granules must still be present, which gelatinize when treated above the gelatinization temperature and cause an additional increase in water content. This is confirmed by the SEM in Figure 1c. Here, starch grains that have not yet gelatinized are partially visible. However, hydration for 5 h and 24 h showed no significance and did not further increase the WAI of PAF samples. After 24 h, a minimal decrease in WAI is observed at all temperatures, which can be attributed to a time‐dependent hysteresis (Barrera et al., 2013). The WAI is mainly affected by the interaction of intra‐ and intermolecular bonds within the amorphous and crystalline structures. Due to the fact that the starch was damaged or gelatinized by puffing, the crystalline structure was disturbed by a break in the inter‐ and intramolecular hydrogen bonds. This leads to more exposed hydroxyl groups for hydrogen bond formation with water (Liu et al., 2017). It can be assumed that these bonds were no longer fully maintained by a longer swelling time, resulting in a slight decrease in WAI after 5 h and 24 h.

**TABLE 1:** Water absorption index (WAI), delta E and yellowness index (YI) of suspensions of puffed amaranth flour (PAF) and raw amaranth flour (AF) suspensions treated at 20, 50 and 80°C for 1, 5, and 24 h

Sample	Temperature [°C]	Soaking time [h]	WAI [g_water_/g_sample_]	∆E	YI
PAF	20	1	5.89 ± 0.21^a^	2.75 ± 0.31^a^	40.23 ± 1.15^a^
	20	5	4.65 ± 0.15^b^	2.60 ± 0.25^a^	40.32 ± 0.55^a^
	20	24	4.49 ± 0.19^c^	2.84 ± 0.35^a^	39.33 ± 0.56^abc^
	50	1	5.98 ± 0.16^ad^	3.92 ± 0.71^b^	38.48 ± 2.05^abc^
	50	5	4.83 ± 0.29^c^	4.33 ± 0.59^b^	38.25 ± 0.64^ab^
	50	24	4.20 ± 0.23^c^	4.66 ± 0.27^b^	38.59 ± 1.24^ab^
	80	1	6.77 ± 0.12^d^	6.03 ± 0.40^c^	38.13 ± 0.95^ab^
	80	5	4.98 ± 0.99^c^	5.71 ± 0.46^c^	38.99 ± 0.46^ab^
	80	24	4.18 ± 0.79^c^	7.08 ± 0.70^d^	37.63 ± 1.26^bc^
AF	20	1	1.41 ± 0.02^r^	3.52 ± 0.71^ru^	18.24 ± 2.82^r^
	20	5	1.27 ± 0.03^r^	4.74 ± 0.49^rs^	12.08 ± 1.43^s^
	20	24	1.27 ± 0.06^r^	3.55 ± 0.80^rstu^	12.75 ± 0.37^su^
	50	1	1.49 ± 0.04^r^	3.58 ± 0.88^rst^	11.92 ± 1.57^stu^
	50	5	1.43 ± 0.06^r^	4.76 ± 0.59^rstu^	12.32 ± 1.88^rstu^
	50	24	1.40 ± 0.02^r^	5.04 ± 0.39^rst^	14.99 ± 0.43^rstu^
	80	1	3.73 ± 0.11^r^	15.92 ± 0.81^rsu^	8.30 ± 0.93^rstu^
	80	5	3.13 ± 0.22^r^	20.28 ± 0.98^ru^	12.34 ± 0.57^rsu^
	80	24	6.16 ± 0.90^s^	20.36 ± 0.42^rv^	16.79 ± 0.71^u^

*Note*: Data are mean values ± SD (*n* = 3).

Mean values in the same column followed by the same letter indicates no significant differences (*p* < .05).

Water absorption index values of AF compared with PAF are significant (*p* < .001). In AF samples, especially at 20°C and 50°C, the amaranth starch was present in a crude form. The WAI was significantly lower compared to PAF. However, the AF samples at 20°C and 50°C showed no significant effect on WAI (*p* = .952). Comparison between treatment times showed that WAI increased significantly at 80°C (*p* > .001), which was due to the gelatinization of raw amaranth starch and protein denaturation (Dayakar Rao et al., 2016). The results of PAF and AF samples are in agreement with those of Gamel et al. (2006) and Burgos and Armada (2015), who found that PAF had a higher WAI compared to AF. The WAI of cooked amaranth flour in the study of Gamel et al. (2006) and the values of AF puffed at 80°C for 1 h in this study showed similar values. Gamel and colleagues also found that puffing was the most effective method for increasing the water absorption of amaranth flour. Mariotti et al. (2006) additionally found that puffing strongly and rapidly increased WAI compared to raw grains. This is partly due to the spongy structure of the matrix and the starch granules gelatinized by puffing. This can also be assumed for the results of this study. Onyango et al. (2020) found that nonstarch polysaccharides have a higher water binding capacity than starch. It is assumed that the nonstarch polysaccharides in AF and PAF also lead to a water increase.

Based on the studies of PAF and AF, the WAI of the blends was investigated at 1 h and 80°C, shown in Table 2. At this temperature, it was found that the WAI of individual PAF and AF samples assumed the highest values, mainly due to additional starch gelatinization of raw starch granules. The WAI of the blends increased with decreasing AF content. Thus, AF contents from 10% to 50% resulted in a higher WAI, also compared to the 100% PAF sample. And the AF/PAF 10/90 sample had the highest WAI with 8.53 g_water_/g_sample_. Islas‐Rubio et al. (2014) attributed the degree of WAI to differences in chemical and protein composition, particle size distribution between samples, degree of starch damage, and gelatinization. Proteins and starch have a high affinity for water, as evidenced by rapid absorption. The hydration kinetics of PAF was faster, which can be attributed to the thermally induced damage of proteins and starch and the homogenous particle size distribution. In the case of AF, water absorption is dependent on temperature. Due to the gelatinization of starch and denaturation of the proteins taking place at 80°C, the water absorption also changes. By comparison, however, the effect was less pronounced for AF. As the proportion of AF increases from 60% to 100%, the WAI values decrease continuously. The lowest WAI was exhibited by the AF‐only sample. The PAF proportion had a significant effect on WAI. De la Barca et al. (2010) also found an increase in WAI with increasing PAF content. They noted that the increased WAI of puffed cereals can be interpreted as starch‐water‐protein interactions. These interactions affected the solid phase structure of the processed material. The WAI of the suspension is then attributed to the gelatinization of starch in excess water. This suspension is enhanced by the degree of starch damage due to gelatinization and fragmentation during processing (de la Barca et al., 2010). It is possible that AF had a positive effect on the cold swelling properties of PAF due to starch gelatinization.

**TABLE 2:** Water absorption index (WAI), delta E and yellowness index (YI) with blends of raw amaranth flour (AF) and puffed amaranth flour (PAF) suspensions treated at 80°C for 1 h

Sample	WAI [g_water_/g_sample_]	∆E	YI
AF/PAF 0/100	6.77 ± 0.12^a^	21.53 ± 0.20^a^	12.75 ± 0.30^a^
AF/PAF 10/90	8.53 ± 0.31^b^	19.33 ± 0.34^ab^	14.55 ± 0.58^abc^
AF/PAF 20/80	7.94 ± 0.32^bc^	18.63 ± 0.33^ac^	20.03 ± 0.83^ac^
AF/PAF 30/70	8.09 ± 0.18^d^	17.13 ± 0.85^ad^	25.12 ± 0.70^acd^
AF/PAF 40/60	7.32 ± 0.24^de^	12.94 ± 1.74^bcde^	27.13 ± 0.70^acde^
AF/PAF 50/50	7.11 ± 0.55^f^	11.97 ± 0.26^bcdf^	30.89 ± 0.55^acd^
AF/PAF 60/40	5.93 ± 0.32^fg^	10.17 ± 1.10^df^	32.02 ± 2.89^ad^
AF/PAF 70/30	5.91 ± 0.11^g^	8.94 ± 0.31^ef^	38.18 ± 2.44^abd^
AF/PAF 80/20	4.67 ± 0.26^gh^	9.45 ± 0.54^ef^	40.95 ± 0.52^ab^
AF/PAF 90/10	4.79 ± 0.06^gh^	7.50 ± 0.32^g^	39.78 ± 1.95^e^
AF/PAF 100/0	3.73 ± 0.11^f^	‐	38.13 ± 0.95^e^

*Note*: Values are given as mean ± SD (*n* = 3).

Means in the same column followed by the same letter indicates no significant differences (*p* < .05).

### 3.4 Color change of amaranth suspensions

Color values (∆E and YI) of all prepared samples are shown in Tables 1 and 2. The PAF samples exhibited a slight color difference ∆E, which, however, increased with increasing temperature. Raw PAF samples showed a light brownish color after grinding, which is homogenous, and the color particles are evenly distributed. During hydration, the colors of PAF suspensions were not significantly affected by temperature and time. Significantly higher ∆E values are shown by the samples treated at 80°C for 5 h and 24 h compared to the other AF samples (*p* < .05). The browning reaction of PAF during the puffing process and of AF samples at 80°C hydration is also mainly due to Maillard reactions and caramelization processes, in which, among others, lysine and other amino acids present react with reducing sugars, which is favored by the processing conditions and leads to a dark coloration (Burgos & Armada, 2015). Abhiram (2018) and Mariotti et al. (2006) found a similar browning reaction when rice was gelatinized with far‐infrared heating. However, PAF samples showed lower sensitivity to color changes. YI is an important parameter for the characterization of pasta, for example (Debbouz & Doetkott, 1996). PAF samples showed a significant difference for temperature (*p* < .001), but no significance for time (*p* = .248) for YI. For the AF samples, both temperature (*p* < .001) and time (*p* < .001) were significant variables affecting YI. There was a significant difference in YI between the PAF and AF samples (*p* < .001). For the AF samples, temperature had a stronger effect on YI. For the 1 h AF samples, the YI decreased with increasing treatment temperature, as it did for the 20°C samples at all treatment times. For the 50°C samples, no significant change in YI occurred within the times studied. Due to starch gelatinization, a Maillard reaction occurred in the 80°C samples, causing a brownish color change and an increase in YI (Burgos & Armada, 2015). Tayefe et al. (2020) also found that heat treatment reduced L* and b* values. Visually, the following can be observed: the AF samples were slightly yellowish, while the PAF samples were brownish.

The ∆E of blends were calculated vs. the AF sample. The AF/PAF blends showed an increasing ∆E with an increase in PAF content, and the YI of blends increased with an increase in AF content. Both effects can be explained by the darker and browner color of PAF compared to AF. Thus, when the PAF content increases, the color value of the samples decreases. Visually, they become increasingly brownish.

The color of food is an important sensory factor (Serna‐Saldivar, 2012). When processing AF and PAF suspensions, it should be noted that the color of PAF suspensions responds to different temperatures and hydration times without much variation. However, PAF suspensions have a darker color than AF suspensions, which can lead to color deviations when used in food processing (e.g., pasta or bread).

### 3.5 Rheological properties

**FIGURE 3:**
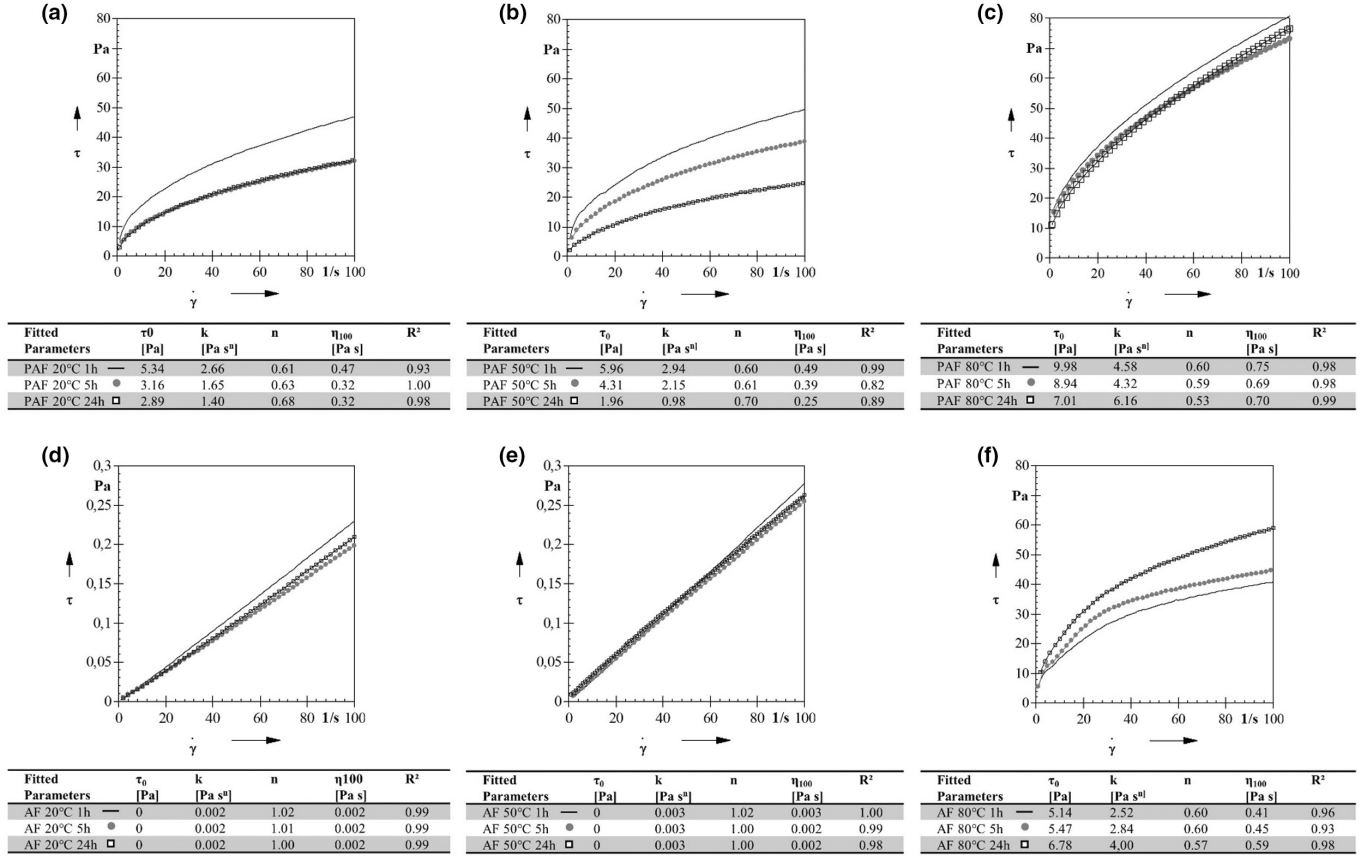
(a–f) Shear stress curves, calculated Hershel‐Bulkley regression parameters and final viscosity at 100 s^−1^ for puffed amaranth flour suspensions (PAF) at (a) 20°C, (b) 50°C, (c) 80°C, and for raw amaranth flour suspensions (AF) at (d) 20°C, (e) 50°C, and (f) 80°C, treated for 1, 5, and 24 h. *τ_0_*, yield stress (Pa); *k*, consistency coefficient (Pa s^nN^); *N*, flow index (dimensionless); η_100_, final viscositsy at =100 1/s and *r^2^*, regeression

The shear stress and shear rate curves and the fitted parameters according to the Herschel‐Bulkley model for the PAF and AF suspensions are shown in Figure 3. The different plots a–c and d–f were treated at 20, 50, and 80°C, respectively. Three different treatment times (1, 5, and 24 h) at a given temperature are also shown within each diagram. The table below each graph shows the mathematical regression *r* ^2^, *n*‐ and *k*‐values, and the final viscosity η_100_ at $\dot{\gamma }$=100 1/s for each measurement.

The results showed that PAF suspensions are nonlinear and required a small force to initiate flow at 20, 50, and 80°C and AF at 80°C at all time points studied. This indicates that the systems behave like non‐Newtonian, shear‐thinning fluids that exhibit yield stress (Figure 3a–c,f), and confirms the SEM images (Figure 2a–d). The *n*‐values determined for all PAF samples were ≤1.00, confirming the assumption of a shear‐thinning fluid. Moreover, the *n*‐values of the PAF samples were not significantly affected by increasing the temperature or time (Figure 3a–c).

The AF suspensions exhibited Newtonian behavior at 20°C and 50°C at all times (Figure 3d,e). The *n*‐values of the AF samples hydrothermally treated at 20°C and 50°C (Figure 3d,f) were 1.00, indicating a Newtonian fluid (τ_0_ = 0.00). Consequently, the *n*‐values for the AF samples at 80°C changed from a Newtonian fluid to a Herschel‐Bulkley fluid (*n* < 1.00). Figure 3c,f shows that the PAF and AF samples treated at 80°C have the highest shear stress due to the higher consistency coefficient, a synonym for viscosity. This is attributed to gelatinization of starch, which in amaranth begins at about 62°C to 68°C (Gamel et al., 2005; Srichuwong et al., 2017), leading to an increase in shear stress and viscosity (Mezger, 2012). The viscosity depends mainly on the collision and shear of the swollen granules. As the granules swell with the degree of gelatinization, the viscosity increases, which is mainly the case at 80°C for AF and PAF samples (Fennema, 1976). However, starch gelatinization was more intense for the AF samples, as indicated by the higher *k*‐value or viscosity. An increase in viscosity was also observed for PAF at 80°C and correlated with the data collected for WAI (*r* = 0.89) and SEM (Table 1, Figure 2b). However, the *k*‐ and η_100_‐values increase significantly at 80°C for PAF and AF. Nevertheless, a difference can be observed between all PAF and AF samples at 80°C. The *n*‐, *k*‐ and the η_100_‐values for the PAF samples were significantly higher due to gelatinization that had already occurred during puffing. Barrera et al. (2013) found that cold swelling of damaged starch granules caused an increase in viscosity even without a temperature influence. This is inconsistent with this study. The η_100_‐values for the PAF samples continued to increase with increasing temperature. The most significant increase was observed for the PAF samples, treated at 80°C. The additional gelatinization of starch due to temperature further increased the viscosity. From the SEM images in Figure 1c, it can be seen that even in the case of PAF, raw starch granules are still present, which are completely gelatinized at 80°C. This resulted in an additional viscosity increase. However, the PAF samples at 20°C and 50°C showed a decrease in *k*‐ and η_100_‐values at longer treatment times. In addition, the viscosity at η_100_ was lower for all PAF samples treated for 5 h and 24 h than for the 1 h samples. This is in agreement with the results obtained at WAI (*r* = 0.97). These observations are probably due to the more advanced hydrolytic cleavage. However, the AF samples at 80°C showed an increase in viscosity with increasing time. The decrease in viscosity with increasing time is due to hysteresis. Barrera et al. (2013) found that hysteresis depends on the content of damaged starch: the higher the content of damaged starch, the higher the hysteresis area. They also described that the fluid structure is time‐dependent as the content of damaged starch increases. This is in consistency with the results of the present study. Damaged or gelatinized retrograded starch is present in PAF, which is due to the puffing process. Considering time, a decrease in viscosity has occurred (Figure 3a–c). Raw amaranth starch is present in the AF samples. This gelatinizes at a temperature of 80°C. Again, an increase in viscosity with time can be seen.

The *k*‐values of the AF samples at 80°C showed a significant increase. According to Barrera et al. (2013), gelatinized starch dispersions prepared under different time and temperature conditions behave like non‐Newtonian fluids, which can exhibit yield stress, as can be seen in the present study. In general, the shear‐thinning flow behavior is attributed to the enlargement of the structural units due to the influence of shear force (Christianson & Bagley, 1984). Starch suspensions during heating have been investigated by Mezger (2012) and Christianson and Bagley (1984). These suspensions changed from a shear‐thickening to a shear‐thinning flow behavior with increasing temperature and time. However, Christianson and Bagley (1984) also found that this behavior was dependent on the starch concentration. The transition from continuous to shear‐thickening behavior occurs only at concentrations above 50% (Crawford et al., 2013). In this study, the suspensions were treated with excess water, which explains the flow behavior of the AF samples: at temperatures below the gelatinization temperature (present study: 20°C and 50°C), the suspensions behaved like Newtonian fluids. If insufficient water is added to the starch suspensions, they behave like shear‐thickening fluids (Mezger, 2012). Only when starch gelatinized did the viscosity increase significantly. In the PAF samples, there was an increase in viscosity immediately after water was added. This is due to the already damaged or gelatinized starch resulting from puffing. This starch can also swell in cold water, which is due to the method of heat treatment (Barrera et al., 2013; Gamel et al., 2006).

**FIGURE 4:**
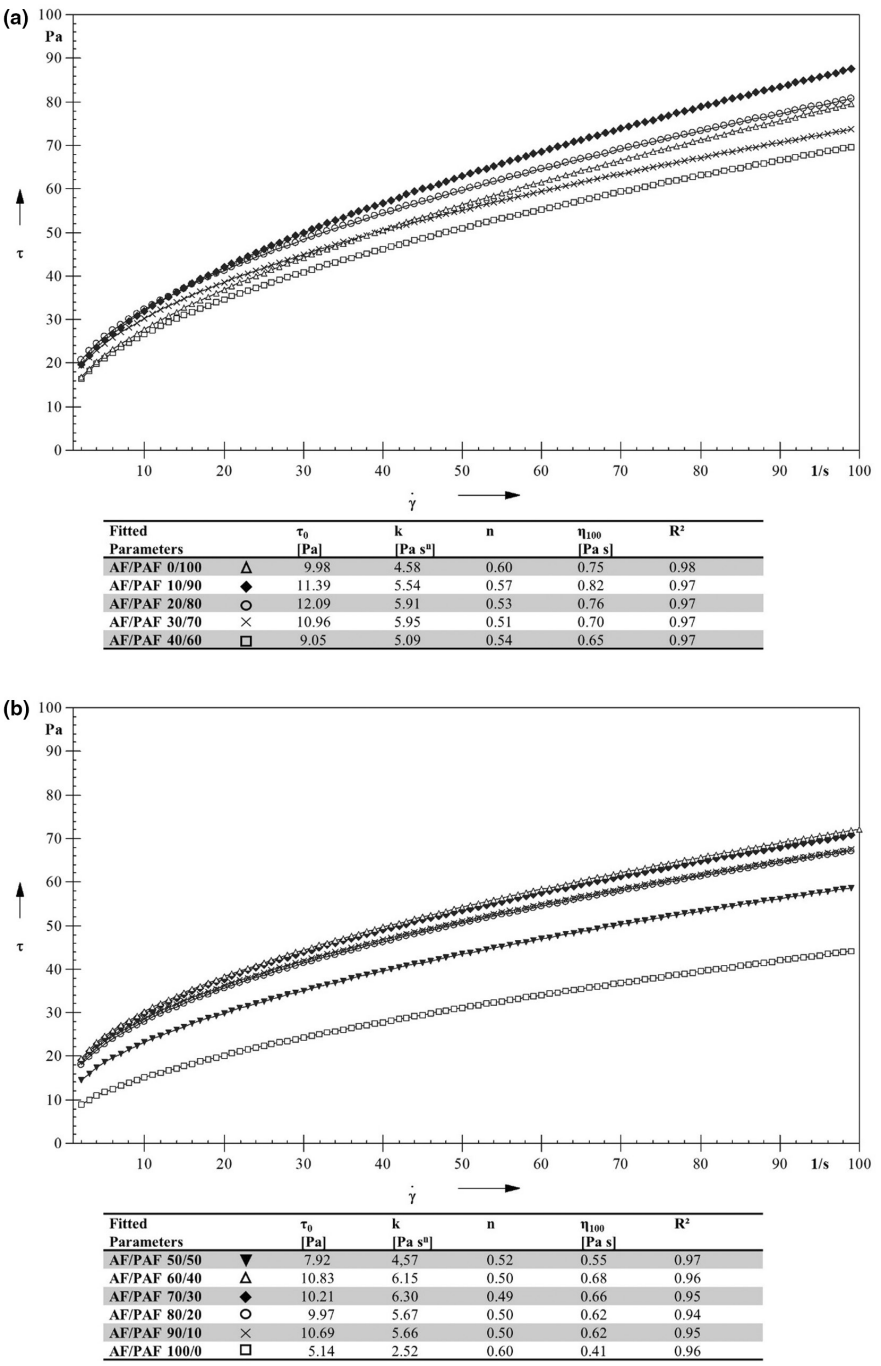
Shear stress curve and calculated Herschel‐Bulkley regression parameters of different blends of Raw Amaranth Flour (AF) to Puffed Amaranth Flour (PAF) from (a) 0 to 40% AF/100 to 60% PAF, and from (b) 50 to 100% AF/50 to 0% PAF, all treated at 80°C for 1 h. *τ_0_*, yield stress (Pa); *k*, consistency coefficient (Pa s^n^); *n*, flow index (dimensionless); η_100_, final viscositsy at =100 1/s and *r^2^*, regeression

The shear rate curves of AF/PAF blends are shown in Figure 4a,b and show non‐Newtonian behavior. The parameters obtained from mathematical curve fitting and the final viscosity η_100_ are also shown below. The *n*‐values ranged from 0.49 to 0.60, but the 0/100 and 100/0 AF/PAF‐sample had the highest *n*‐values. The 100/0 AF/PAF‐sample also had the lowest viscosity, expressed by the *k*‐ and η_100_‐values of all samples. The *k‐* and η_100_‐values of the blends were strongly dependent on the composition of AF/PAF. The viscosity of the AF/PAF mixtures decreased with decreasing PAF content, up to the mixture 50/50 AF/PAF. In this blend, relatively low *k*‐ and η_100_‐value were observed. Subsequently, the *k*‐values and viscosity increased again with increasing AF content. In the blend 50/50 AF/PAF, an inflection point is reached in the shear‐dependent behavior of the blends. For the blends with an increased PAF content of >50%, the blends are largely determined by the water binding of the PAF. For the blends with an increased AF content of >50%, the viscosity and the behavior of the blend are mainly determined by the starch gelatinization of the AF.

These results show that the mixtures of AF and PAF influence the properties of the suspension. The higher the PAF or AF content, the higher the viscosity. However, PAF was also found to have a greater effect on viscosity than AF.

## 4 CONCLUSION

The results show that the flours of raw amaranth flour (AF) and puffed amaranth flour (PAF) differ in the properties of their suspensions. Puffing significantly influences the amaranth suspensions regarding an increase of viscosity and water absorption and a decrease in color and yellowness index. The puffing process causes damage to the starch granules within the amaranth grain. As a result, their properties change and swell, for example, in cold water, as in this study. This is particularly evident in the fact that in the cold state, PAF suspensions already have a non‐Newtonian, shear‐thinning flow character, while AF suspensions show Newtonian flow properties. Their flow character is transformed only by heating to the gelatinization temperature. Temperature effects, however, influenced the studied factors more than time: an increase in viscosity and WAI was observed for both AF and PAF at a temperature treatment above the gelatinization temperature.

However, for the blends of PAF and AF, PAF was found to have a stronger effect on viscosity. It was also determined that a small admixture of PAF increased the viscosity of AF above the gelatinization temperature. Increasing the amount of PAF in the blend also resulted in an increase in WAI and a decrease in color value.

Puffed amaranth flours suspensions and PAF/AF blends can be used in various food applications. They can help to increase the viscosity of doughs, for example, to produce gluten‐free foods.

## Data Availability

The data that support the findings of this study are available from the corresponding author upon reasonable request.

## References

[fsn32970-bib-0001] Abhiram, G. (2018). The correlation of colour and viscosity changes of rice flour with gelatinization percentage under infrared heating. International Journal of Research and Review, 5(2), 5. 10.4444/ijrr.1002/514

[fsn32970-bib-0002] Alonso‐Miravalles, L. , Zannini, E. , Bez, J. , Arendt, E. K. , & O'Mahony, J. A. (2020). Physical and flow properties of pseudocereal‐based protein‐rich ingredient powders. Journal of Food Engineering, 281, 109973. 10.1016/j.jfoodeng.2020.109973

[fsn32970-bib-0003] Amare, E. , Mouquet‐Rivier, C. , Servent, A. , Morel, G. , Adish, A. , & Haki, G. D. (2015). Protein quality of amaranth grains cultivated in Ethiopia as affected by popping and fermentation. Food and Nutrition Sciences, 6(1), 38–48. 10.4236/fns.2015.61005

[fsn32970-bib-0004] Barbosa‐Cánovas, G. , Ortega‐Rivas, E. , Juliano, P. , & Yan, H. (2005). Food powders: Physical properties, processing, and functionality (p. 382). Springer.

[fsn32970-bib-0005] de la Barca, A. M. , Rojas‐Martinez, M. E. , Islas‐Rubio, A. R. , & Cabrera‐Chavez, F. (2010). Gluten‐free breads and cookies of raw and popped amaranth flours with attractive technological and nutritional qualities. Plant Foods for Human Nutrition, 65(3), 241–246. 10.1007/s11130-010-0187-z 20734143

[fsn32970-bib-0006] Barrera, G. N. , Bustos, M. C. , Iturriaga, L. , Flores, S. K. , León, A. E. , & Ribotta, P. D. (2013). Effect of damaged starch on the rheological properties of wheat starch suspensions. Journal of Food Engineering, 116(1), 233–239. 10.1016/j.jfoodeng.2012.11.020

[fsn32970-bib-0007] Bressani, R. (1994). Composition and nutritional properties of amaranth. In O. Paredes‐Lopez (Ed.), Amaranth biology, chemistry, and technology (p. 2334). CRC Press.

[fsn32970-bib-0008] Burgos, V. E. , & Armada, M. (2015). Characterization and nutritional value of precooked products of kiwicha grains (*Amaranthus caudatus*). Food Science and Technology (Campinas), 35(3), 531–538. 10.1590/1678-457x.6767

[fsn32970-bib-0009] Burgos, V. E. , & Armada, M. (2021). Microstructure and storage stability of precooked kiwicha products. Journal of Food Processing and Preservation, 45(11), e15911. 10.1111/jfpp.15911

[fsn32970-bib-0010] Burgos, V. E. , Binaghi, M. J. , de Ferrer, P. A. R. , & Armada, M. (2018). Effect of precooking on antinutritional factors and mineral bioaccessibility in kiwicha grains. Journal of Cereal Science, 80, 9–15. 10.1016/j.jcs.2017.12.014

[fsn32970-bib-0011] Castro‐Giráldez, M. , Fito, P. J. , Prieto, J. M. , Andrés, A. , & Fito, P. (2012). Study of the puffing process of amaranth seeds by dielectric spectroscopy. Journal of Food Engineering, 110(2), 298–304. 10.1016/j.jfoodeng.2011.04.012

[fsn32970-bib-0012] Christianson, D. D. , & Bagley, E. B. (1984). Yield stresses in dispersions of swollen, deformable cornstarch granules. Cereal Chemistry, 61, 4.

[fsn32970-bib-0013] Crawford, N. C. , Popp, L. B. , Johns, K. E. , Caire, L. M. , Peterson, B. N. , & Liberatore, M. W. (2013). Shear thickening of corn starch suspensions: Does concentration matter? Journal of Colloid and Interface Science, 396, 83–89. 10.1016/j.jcis.2013.01.024 23484772

[fsn32970-bib-0014] Dayakar Rao, B. , Anis, M. , Kalpana, K. , Sunooj, K. V. , Patil, J. V. , & Ganesh, T. (2016). Influence of milling methods and particle size on hydration properties of sorghum flour and quality of sorghum biscuits. LWT ‐ Food Science and Technology, 67, 8–13. 10.1016/j.lwt.2015.11.033

[fsn32970-bib-0015] Debbouz, A. , & Doetkott, C. (1996). Effect of process variables on spaghetti quality. Cereal Chemistry, 73(6), 5.

[fsn32970-bib-0016] Fennema, O. R. (1976). Food chemistry. M. Dekker.

[fsn32970-bib-0017] Gamel, T. H. , Linssen, J. P. , Mesallam, A. S. , Damir, A. A. , & Shekib, L. A. (2006). Seed treatments affect functional and antinutritional properties of amaranth flours. Journal of the Science of Food and Agriculture, 86(7), 1095–1102. 10.1002/jsfa.2463

[fsn32970-bib-0018] Gamel, T. H. , Linssen, J. P. , Mesallem, A. S. , Damir, A. A. , & Shekib, L. A. (2005). Effect of seed treatments on the chemical composition and properties of two amaranth species: Starch and protein. Journal of the Science of Food and Agriculture, 85(2), 319–327. 10.1002/jsfa.1988

[fsn32970-bib-0019] González, R. J. , Torres, R. L. , Greef, D. M. D. , Tosi, E. , & Re, E. (2002). Effects of popping and extrusion processes on some hydration properties of amaranth. Brazilian Journal of Chemical Engineering, 19(4), 391–395. 10.1590/S0104-66322002000400006

[fsn32970-bib-0020] Hidalgo, A. , Scuppa, S. , & Brandolini, A. (2016). Technological quality and chemical composition of puffed grains from einkorn (triticum monococcum l. Subsp. Monococcum) and bread wheat (triticum aestivum l. Subsp. Aestivum). LWT ‐ Food Science and Technology, 68, 541–548. 10.1016/j.lwt.2015.12.068

[fsn32970-bib-0021] Islas‐Rubio, A. R. , Calderón de la Barca, A. M. , Cabrera‐Chávez, F. , Cota‐Gastélum, A. G. , & Beta, T. (2014). Effect of semolina replacement with a raw: Popped amaranth flour blend on cooking quality and texture of pasta. LWT ‐ Food Science and Technology, 57(1), 217–222. 10.1016/j.lwt.2014.01.014

[fsn32970-bib-0022] Konishi, Y. , Iyota, H. , Yoshida, K. , Moritani, J. , Inoue, T. , Nishimura, N. , & Nomura, T. (2004). Effect of moisture content on the expansion volume of popped amaranth seeds by hot air and superheated steam using a fluidized bed system. Bioscience, Biotechnology, and Biochemistry, 68(10), 2186–2189. 10.1271/bbb.68.2186 15502367

[fsn32970-bib-0023] Lara, N. , & Ruales, J. (2002). Popping of amaranth grain (amaranthus caudatus) and its effect on the functional, nutritional and sensory properties. Journal of the Science of Food and Agriculture, 82(8), 797–805. 10.1002/jsfa.1069

[fsn32970-bib-0024] Liu, Y. , Chen, J. , Luo, S. , Li, C. , Ye, J. , Liu, C. , & Gilbert, R. G. (2017). Physicochemical and structural properties of pregelatinized starch prepared by improved extrusion cooking technology. Carbohydrate Polymers, 175, 265–272. 10.1016/j.carbpol.2017.07.084 28917865

[fsn32970-bib-0025] Lux, T. , Wernecke, C. , Bosse, R. , Reimold, F. , & Flöter, E. (2021). Textural and morphological changes of heat soaked raw amaranthus caudatus. Journal of Cereal Science, 98, 103168. 10.1016/j.jcs.2021.103168

[fsn32970-bib-0026] Mariotti, M. , Alamprese, C. , Pagani, M. A. , & Lucisano, M. (2006). Effect of puffing on ultrastructure and physical characteristics of cereal grains and flours. Journal of Cereal Science, 43(1), 47–56. 10.1016/j.jcs.2005.06.007

[fsn32970-bib-0027] Martinez‐Villaluenga, C. , Penas, E. , & Hernandez‐Ledesma, B. (2020). Pseudocereal grains: Nutritional value, health benefits and current applications for the development of gluten‐free foods. Food and Chemical Toxicology, 137, 111178. 10.1016/j.fct.2020.111178 32035214

[fsn32970-bib-0028] Mezger, T. (2012). Das Rheologie Handbuch (4th ed.). Hannover Vincentz Network.

[fsn32970-bib-0029] Onyango, C. , Luvitaa, S. K. , Unbehend, G. , & Haase, N. (2020). Physico‐chemical properties of flour, dough and bread from wheat and hydrothermally‐treated finger millet. Journal of Cereal Science, 93, 102954. 10.1016/j.jcs.2020.102954

[fsn32970-bib-0030] Paucar‐Menacho, L. M. , Dueñas, M. , Peñas, E. , Frias, J. , & Martínez‐Villaluenga, C. (2018). Effect of dry heat puffing on nutritional composition, fatty acid, amino acid and phenolic profiles of pseudocereals grains. Polish Journal of Food and Nutrition Sciences, 68(4), 289–297. 10.1515/pjfns-2018-0005

[fsn32970-bib-0031] Rao, M. A. (2007). Rheology of food gum and starch dispersions. In M. A. Roa (Ed.), Rheology of solid and semisolid foods (Vol. 2, p. 491). Springer.

[fsn32970-bib-0032] Rufián‐Henares, J. Á. , Guerra‐Hernandez, E. , & García‐Villanova, B. (2006). Colour measurement as indicator for controlling the manufacture and storage of enteral formulas. Food Control, 17(6), 489–493. 10.1016/j.foodcont.2005.02.011

[fsn32970-bib-0033] Schmidt, D. , Verruma‐Bernardi, M. R. , Forti, V. A. , & Borges, M. T. M. R. (2021). Quinoa and amaranth as functional foods: A review. Food Reviews International, 1‐20. 10.1080/87559129.2021.1950175

[fsn32970-bib-0034] Schoenlechner, R. (2017). Pseudocereals in gluten‐free products. In C. M. Haros & R. Schoenlechner (Eds.), Pseudocereals: Chemistry and technology (Vol. 1, pp. 193–216). John Wiley & Sons, Ltd.

[fsn32970-bib-0035] Schwenke, K. D. , Prahl, L. , Rauschal, E. , Gwiazda, S. , Dabrowski, K. , & Rutkowski, A. (1981). Functional properties of plant proteins. Part 2. Selected physicochemical properties of native and denatured protein isolates form fava beans, soybeans and sunflower seed. Die Nahrung, 1, 11.

[fsn32970-bib-0036] Serna‐Saldivar, S. O. (2012). Determination of color, texture and sensory properties of cereal grain products. In C. Press (Ed.), Cereal grains‐ laboratory reference and procedures manual (Vol. 1, pp. 91–105). Taylor & Francis Inc.

[fsn32970-bib-0037] Singh, N. , Kaur, S. , Kaur, A. , Isono, N. , Ichihashi, Y. , Noda, T. , & Rana, J. C. (2014). Structural, thermal, and rheological properties ofamaranthus hypochondriacus and amaranthus caudatus starches. Starch ‐ Stärke, 66(5–6), 457–467. 10.1002/star.201300157

[fsn32970-bib-0038] Singh, M. , & Sood, S. (2021). In M. Singh & S. Sood (Eds.), Millets and pseudo cereals. Woodhead Publishing.

[fsn32970-bib-0039] Srichuwong, S. , Curti, D. , Austin, S. , King, R. , Lamothe, L. , & Gloria‐Hernandez, H. (2017). Physicochemical properties and starch digestibility of whole grain sorghums, millet, quinoa and amaranth flours, as affected by starch and non‐starch constituents. Food Chemistry, 233, 1–10. 10.1016/j.foodchem.2017.04.019 28530552

[fsn32970-bib-0040] Tayefe, M. , Shahidi, S.‐A. , Milani, J. M. , & Sadeghi, S. M. (2020). Development, optimization, and critical quality characteristics of new wheat‐flour dough formulations fortified with hydrothermally‐treated rice bran. Journal of Food Measurement and Characterization, 14(5), 2878–2888. 10.1007/s11694-020-00532-y

